# Nationwide Observational Case–Control Study of Risk Factors for *Aerococcus* Bloodstream Infections, Sweden

**DOI:** 10.3201/eid3105.240424

**Published:** 2025-05

**Authors:** John Walles, Malin Inghammar, Magnus Rasmussen, Torgny Sunnerhagen

**Affiliations:** Central Hospital Kristianstad, Kristianstad, Sweden (J. Walles); Lund University, Malmö, Sweden (J. Walles); Lund University, Lund, Sweden (J. Walles, M. Inghammar, M. Rasmussen, T. Sunnerhagen); Skåne University Hospital, Lund (J. Walles, M. Inghammar, M. Rasmussen, T. Sunnerhagen)

**Keywords:** *Aerococcus*, bacteremia, case-control studies, risk factors, sepsis, urinary tract infection, neurologic conditions, bacteria, Sweden

## Abstract

Risk factors for developing bloodstream infections (BSIs) caused by *Aerococcus* bacteria remain insufficiently examined. In this nationwide case–control study in Sweden, 19 of 23 clinical microbiological laboratories identified patients who had aerococcal BSIs during 2012–2016. We compared each of those index patients with 4 controls matched for age, sex, and county of residence. Overall, 588 episodes of aerococcal BSI occurred over 39.6 million person-years, corresponding to an average incidence of 1.48/100,000 person-years (95% CI 1.37–1.60/100,000 person-years). Most infections developed in men >65 years of age. Aerococcal BSI was associated with neurologic (adjusted odds ratio 2.89 [95% CI 2.26–3.70]) and urologic (adjusted odds ratio 2.15 [95% CI 1.72—2.68]) conditions and previous hospitalization or infection treatment. Our findings support the previously observed predilection for aerococcal BSIs developing in elderly men with urinary tract disorders. Awareness of *Aerococcus* spp. in patients, especially elderly men, will be needed to manage invasive infections.

Invasive infections caused by bacteria of the genus *Aerococcus* have been increasingly recognized; however, because of difficulties in identification during routine care, patients at risk for those infections have been insufficiently examined. *Aerococcus* bacteria were first described in 1953 (*1*), identified as relatives of bacteria belonging to *Streptococcus* and *Enterococcus* genera. Since then, specific species have been discovered, of which *A. urinae* and *A. sanguinicola* are most commonly observed in human infections (*2*–*4*).

Before matrix-assisted laser desorption/ionization time-of-flight (MALDI-TOF) mass spectrometry was introduced, accurate identification of *Aerococcus* at the species level was difficult. Consequently, the characteristics of aerococcal infections and infected patients were not described extensively until about 2016, primarily through case series reports (*5*–*8*).

*Aerococcus* spp. are a fundamental cause of urinary tract infections (*9*–*11*). However, the rates of severe infections, such as aerococcal bloodstream infections (BSIs), most notably found in elderly men and persons with urinary tract disorders (*6*,*12*–*17*), and aerococcal infective endocarditis have also increased in recent years (*5*,*14*,*17*–*20*). Despite previous studies indicating that most patients with aerococcal infective endocarditis tend to be elderly and have underlying illnesses, the prognosis appears to be favorable (*18*). The increased number of reported cases is likely attributed to MALDI-TOF mass spectrometry, a reliable tool for correctly identifying aerococci and the preferred method for determining *Aerococcus* species in most clinical microbiology laboratories (*7*,*21*). Aerococci have been suggested to adhere to urinary catheters and urothelium and, thus, form biofilms, but the precise mechanisms by which they do so remain unknown (*22*–*24*). Epidemiologic studies have been limited primarily to case series. In this national study, we examined the incidence of and risk factors for aerococcal BSI in Sweden during 2012–2016. This study was approved by the regional ethics review board of Lund University, Sweden (approval no. 2016/938). The retrospective nature of the study obviated the need to obtain informed consent.

## Materials and Methods

### Study Design and Setting

In a retrospective, matched case–control study, we compared a population of persons who had culture-proven aerococcal BSI with a matched control population by using a set of potential predisposing conditions that existed for >1 month before the detection of the BSI. Predisposing conditions were predefined by registered care and drug prescriptions data in national healthcare registers of Sweden. Healthcare in Sweden is publicly financed and provided regardless of a person’s financial or health insurance status. Permanent residents of Sweden are assigned a unique and lifelong 10-digit personal identification number, enabling cross-referencing of health data sources (*25*).

### Retrieval of Cases and Controls

**Figure 1:**
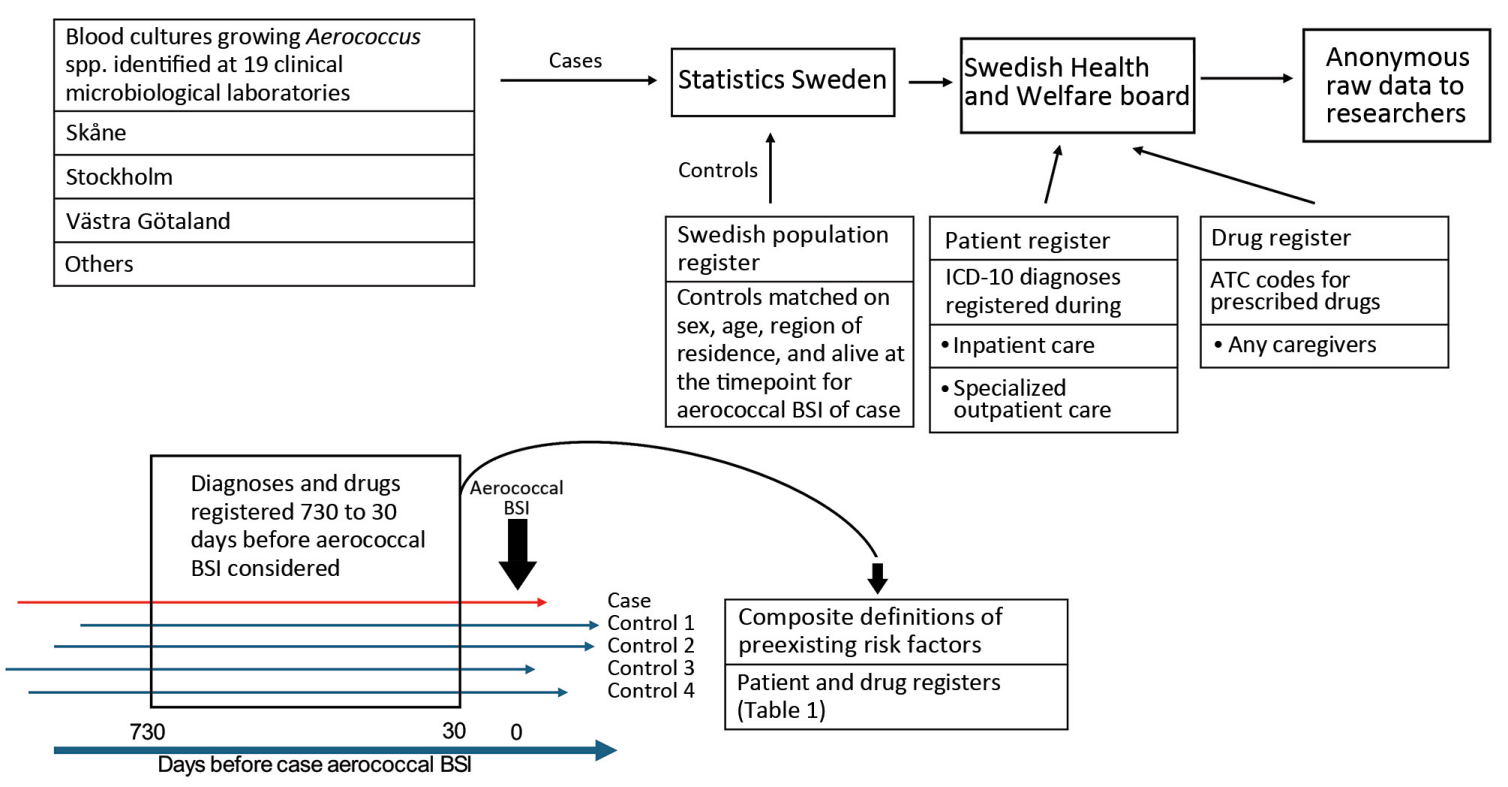
Overview of data collection and curation in nationwide observational case–control study of risk factors for *Aerococcus* BSIs, Sweden. Cases of aerococcal BSI were identified at 19 clinical microbiological laboratories across Sweden during 2012–2016. Matched control data were obtained from the Swedish Population Register. Registered diagnoses were collected from the National Patient Register, and prescribed drug data were collected from the National Drug Register. Registrations performed 30–730 days before aerococcal BSI detection were used to define medical conditions and characteristics hypothesized to contribute to aerococcal BSI. ATC, Anatomic Therapeutic Chemical; BSI, bloodstream infection; ICD-10, International Classification of Diseases, 10th Revision.

Clinical microbiology laboratories (19 of 23) in Sweden identified patients with >1 episode of aerococcal BSI (defined as the detection of *Aerococcus* bacteria in blood culture, regardless of the species or the presence of other bacteria) during 2012–2016; the remaining 4 laboratories declined participation (Figure 1). For each case, Statistics Sweden (https://www.scb.se) selected 4 controls matched for sex, age (+2 years difference), and county of residence by using the Population Register of Sweden (*26*). We required that each person designated as a control had been alive on the date the aerococcal BSI was detected in the corresponding study patient. We intended the controls to reflect a similar distribution of age, sex, and county of residence as the case-patients, irrespective of any interaction with the healthcare system. Sweden’s National Board of Health and Welfare linked study participants to registered diagnosis codes (classified according to the International Classification of Diseases, 10th revision [ICD-10]) from hospital admissions data (National Inpatient Register) and specialized ambulatory care data (National Outpatient Register) (*25*,*27*); diagnoses registered in primary care were not included. The National Board of Health and Welfare also linked study participants to drugs prescribed during specialized and primary healthcare (classified by the Anatomic Therapeutic Chemical classification system) from the National Prescribed Drug Register (*25*). We defined repeat aerococcal BSI episodes as relapses if they occurred within 90 days; we excluded relapse episodes from all analyses. We defined reinfection as an aerococcal BSI occurring >90 days after the index episode; we retained those cases for incidence calculations but excluded them from other analyses.

### Rationale and Definitions of Predisposing Conditions

We defined a set of mechanisms that we considered plausible contributors to the risk for aerococcal BSI: ecologic dysregulation, compromise of local dermal and mucosal barriers, compromise of systemic immune competence, displacement of urogenital or fecal microbiota, healthcare exposure, and frailty (Table 1). Subsequently, we selected a set of specific, defined predisposing conditions to capture >1 of those mechanisms (Table 1). For example, we considered the treatment for infection variable to be relevant for capturing aspects of ecologic dysregulation, healthcare exposure, and compromise of local barriers, whereas we considered diabetes mellitus to capture ecologic dysregulation, immune competence, healthcare exposure, and frailty.

**Table 1:** Hypothesized mechanisms for and operational definitions of participants’ medical conditions in nationwide observational case–control study of risk factors for *Aerococcus* BSIs, Sweden, 2012–2016*

Conditions	Hypothesized mechanisms linking conditions to aerococcal BSI							
	Ecologic dysregulation/displacement	Compromised local barriers	Lowered immune competence	Healthcare exposure	Frailty		Operational definitions	
							Patient register, ICD-10 codes	Drug register, ATC codes
Hospital admissions	No	No	No	**Yes**	**Yes**		Registered admissions	NA
Prescribed drugs	No	No	No	**Yes**	**Yes**		NA	Registered prescriptions
Treatment for infection	**Yes**	**Yes**	No	**Yes**	No		A, B, M00–03, G00–08, I33, I38–40, J0–2, N10	J01
Pulmonary disease	No	**Yes**	No	**Yes**	**Yes**		J3–9	R03
Gastrointestinal disease	**Yes**	**Yes**	No	**Yes**	No		K2–9	NA
Malignant disease, nonurologic	No	**Yes**	Yes	**Yes**	**Yes**		C0–4, C50, C54–58, C69, C7–9, Z923, Z926	NA
Urologic conditions, including malignancy	No	**Yes**	No	No	No		C51–53, C60–68, R3, N2–5, N7–9, Q5, Q60–64, Z935–36	G04c, G04bd, G04bx
Neurologic conditions	**Yes**	No	No	**Yes**	**Yes**		G0–3, G6–8, F0, F7, F8, I6	N06d, N04
Corticosteroid treatment	No	No	Yes	No	No		NA	H02ab, H02aa02, A07ea
Diabetes mellitus	**Yes**	No	**Yes**	**Yes**	**Yes**		E10–14	A10a, A10b
Heart disease	No	No	No	**Yes**	**Yes**		I0, I2–5, I7, Z45, Q2	C03c
Rheumatic disease	No	No	**Yes**	**Yes**	**Yes**		M	NA
Kidney disease	No	No	**Yes**	**Yes**	**Yes**		N0, N11–19	NA

*Condition or characteristic considered to capture aspects of >1 pre hoc hypothesized mechanisms for aerococcal BSI. Bold font indicates positive association. Conditions were defined operationally by the presence of any criteria listed in the final 2 columns (registered diagnosis or drug prescription); absence of a condition was defined as lack of all listed criteria for that condition. Conditions were considered for registrations made 1–24 months (excluding the most recent month) preceding the aerococcal BSI detection or control date. ATC, Anatomic Therapeutic Chemical; BSI, bloodstream infection; ICD-10, International Classification of Diseases, 10th Revision; NA, not applicable.

We included the following variables in multivariable analyses: number of hospital admissions (categorized as 0, 1–2, 3–5, 6–10, or >10), total number of prescribed drugs, pulmonary disease, gastrointestinal disease, malignant disease, structural urologic condition, corticosteroid treatment, rheumatologic disease, chronic kidney disease, neurologic condition, diabetes mellitus, cardiovascular disease, and previous treatment for infection (categorized as a filled prescription for any antimicrobial drug or registered treatment for infectious disease before the index date: 1–3 months, 4–9 months, 10–24 months, >24 months, or never). To capture conditions that were preexisting and relevant at the time of the aerococcal BSI but avoid conditions that were caused or aggravated by the infection, we only considered diagnoses and prescribed drugs registered from 2 years to 30 days before aerococcal BSI detection; the same dates were applied for the matched controls. When no component of the definition for a particular condition was registered, we considered those patients did not have that condition.

### Statistical Analysis

We extracted publicly available data from Statistics Sweden and stratified by age and sex for the total population that inhabited the uptake areas of the participating laboratories during the study period (*28*). We calculated crude, age-stratified, and sex-stratified incidence as the number of cases that occurred within the strata divided by the number of persons within the respective strata of the source population and the study period in years. We included repeat episodes of aerococcal BSI for this analysis if they were separated by >90 days.

We applied conditional logistic regression models to analyze multivariable associations between underlying conditions and aerococcal BSIs, accounting for matching. We selected the variables for the final model by structured backward selection according to likelihood-ratio tests and a p value of <0.10 as the threshold that defined a loss of model fitness after elimination of a variable. For each model, we assessed multicollinearity and used a variance inflation factor threshold of 2.4 to discard variables affected by multicollinearity, even if it led to loss of model fitness.

We performed secondary analyses by using models stratified by sex and age (in tertiles) and presented those data as forest plots. We conducted statistical analyses and generated graphs by using R version 4.3.1 (The R Project for Statistical Computing, https://www.r-project.org). We used the clogit function of the survival package for conditional logistic regression, the lmtest function to perform likelihood-ratio tests, the car function to assess variance inflation, and the forestplot function to construct forest plots.

## Results

Of all 23 clinical microbiology laboratories in Sweden, 19 provided data for this study; those laboratories collectively serve 8.78 million persons and correspond to ≈90% of the country’s population. The laboratories contributed data on cases within a 2- to 5-year period (depending on the time of introduction of MALDI-TOF mass spectrometry for *Aerococcus* spp. identification), yielding a total observation of 39.6 million person-years.

**Figure 2:**
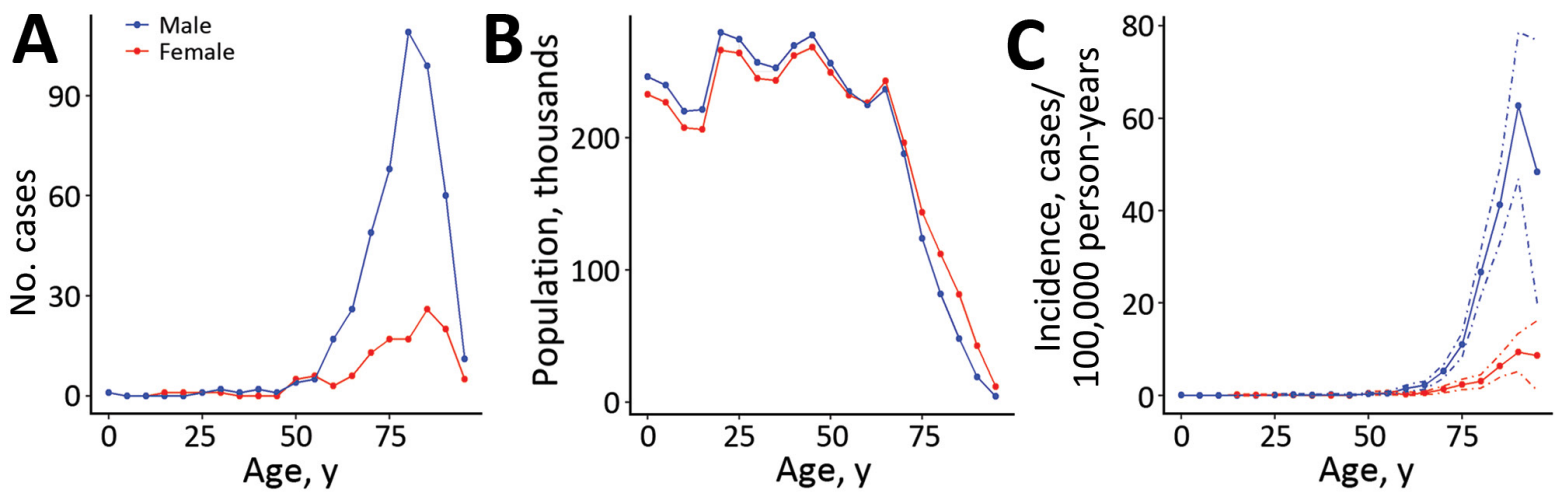
Age and sex distribution of patients in nationwide observational case–control study of risk factors for *Aerococcus* bloodstream infections (BSIs), stratified by age in 5-year intervals and sex, Sweden, 2012–2016. A) Number of patients with aerococcal BSI. B) Populations within the uptake area at risk for aerococcal BSI. C) Incidence of aerococcal BSI.

We identified a total of 591 episodes of aerococcal BSI in 581 persons. Among those episodes, 3 were considered relapses (<90-day interval) and excluded from our incidence calculations. The average incidence of aerococcal BSI was 1.48/100,000 person-years (95% CI 1.37–1.60/100,000 person-years). Most aerococcal BSIs occurred in older persons; the median age was 82 (interquartile range 74–87) years, and 78.6% (452 cases) of infected patients were men (Figure 2, panels A, B). Other than 2 cases that occurred in children <1 year of age, the age-stratified incidence was ≈0 in persons <60 years of age for both sexes, after which a substantial increase in the number of cases occurred, especially in men (maximum incidence 62.8/100,000 person-years [95% CI 46.9–78.6/100,000 person-years] in men 90–94 years of age) (Figure 2, panel C).

Most (n = 422 [71.7%]) aerococcal BSI episodes were caused by *A. urinae*, whereas 61 (10.4%) episodes were attributed to *A. sanguinicola* and 91 (15.5%) episodes were caused by other or unspecified aerococci; 14 (2.4%) patients had 2 concomitant species of aerococci in their blood cultures. Additional species of bacteria were identified in blood cultures from 201 (34.2%) cases, including 62 (10.5%) cultures containing coagulase-negative staphylococci, 35 (6.0%) with *Escherichia coli*, 26 (4.4%) with *Actinotignum schaali*, 17 (2.9%) with *Enterococcus faecalis*, and 15 (2.6%) with *Staphylococcus aureus*.

### Comparison with Matched Controls

We identified 4 matched controls for each of 577 (99.3%) of 581 patients with aerococcal BSIs; the resulting 2,885 persons constituted the study population. Crude analyses revealed significantly higher proportions of most medical conditions in participants with aerococcal BSIs than in controls, including structural urologic (331/577 [57.4%] vs. 753/2308 [31.8%]; p<0.001) and neurologic (227/577 [39.3%] vs. 318/2308 [13.8%]; p<0.001) conditions (Table 2).

**Table 2:** Characteristics of participants in nationwide observational case–control study of risk factors for *Aerococcus* BSIs, Sweden, 2012–2016*

Characteristics	Controls, n = 2,308	Aerococcal BSI, n = 577	p value
Age, y			
Median (IQR)	82 (74–87)	82 (74–87)	1.0
0–5	8 (0.3)	2 (0.3)	
6–18	0	0	
19–76	796 (34.5)	199 (34.5)	
77–85	740 (32.1)	185 (32.1)	
>85	764 (33.1)	191 (33.1)	
Sex			1.0
M	1,816 (78.7)	454 (78.7)	
F	492 (21.3)	123 (21.3)	
Residence, regional council			1.0
Skåne	584 (25.3)	146 (25.3)	
Stockholm	396 (17.2)	99 (17.2)	
Västra Götaland	376 (16.3)	94 (16.3)	
Kalmar	124 (5.4)	31 (5.4)	
Uppsala	100 (4.3)	25 (4.3)	
Other†	728 (31.5)	182 (31.5)	
Previous treatment for another infection,‡ mo			<0.001
1–3	231 (10.0)	129 (22.4)	
>3–9	281 (12.2)	100 (17.3)	
>9–24	416 (18.0)	122 (21.1)	
>24 or never	1,380 (59.8)	226 (39.2)	
Malignant disease, nonurologic	182 (7.9)	75 (13.0)	<0.001
Urologic conditions§	753 (31.8)	331 (57.4)	<0.001
Neurologic conditions	318 (13.8)	227 (39.3)	<0.001
Hospital admissions, median (IQR)	0 (0–1)	1 (0–3)	<0.001
Kidney disease	93 (4.0)	52 (9.0)	<0.001
Pulmonary disease	346 (15.0)	122 (21.1)	0.001
Gastrointestinal disease	255 (11.0)	105 (18.2)	<0.001
Diabetes mellitus	324 (14.0)	113 (19.6)	<0.001
Cardiovascular disease	814 (35.3)	292 (50.6)	<0.001
Number of prescribed drugs, median (IQR)	7 (4–12)	11 (7–16)	<0.001
Rheumatological disease	390 (16.9)	130 (22.5)	0.002
Corticosteroid therapy	309 (13.4)	96 (16.6)	0.05

*Values are no. (%) except as indicated. The matching variables age, sex, and geographic region were distributed equally, but patients with aerococcal BSI were disproportionately affected by all investigated conditions. p values were calculated by χ^2^ or Mann-Whitney U tests as appropriate. BSI, bloodstream infection; IQR, interquartile range. †Other regions were Östergötland, Jönköping, Kronoberg, Blekinge, Halland, Värmland, Örebro, Västmanland, Dalarna, and Norrbotten. ‡Time in months indicates when treatment for another infection occurred before the aerococcal BSI was detected. §Including urologic malignancies.

Structured progressive modeling yielded a final conditional logistic regression model in which aerococcal BSI was associated with structural urologic conditions (adjusted odds ratio [aOR] 2.15 [95% CI 1.72–2.68]; p<0.001), neurologic conditions (aOR 2.89 [95% CI 2.26–3.70]; p<0.001), number of hospital admissions (aOR 6.81 [95% CI 2.51–8.5]; p<0.001 for >10 vs. 0 hospitalizations), and previous treatment for infection (for treatment 1–3 months before infection compared with >2 years or never, aOR 1.88 [95% CI 1.38–2.56]; p<0.001) (Table 3). A borderline significant negative association with rheumatologic diseases was also observed (aOR 0.77 [95% CI 0.59–1.00]; p = 0.05).

**Table 3:** Multivariable models used in nationwide observational case–control study of risk factors for *Aerococcus* BSIs, Sweden, 2012–2016*

Characteristics	First model			Final model	
	Adjusted odds ratio (95% CI)	p value		Adjusted odds ratio (95% CI)	p value
Previous treatment for infection,† mo					
Never	Referent	NA		Referent	NA
1–3	1.77 (1.28–2.45)	<0.001		1.88 (1.38–2.56)	<0.001
>3–9	1.15 (0.83–1.58)	0.40		1.21 (0.89–1.65)	0.23
>9–24	1.19 (0.90–1.59)	0.22		1.22 (0.92–1.61)	0.16
Malignant disease, nonurologic	1.40 (1.01–1.95)	0.046		1.39 (1.00–1.93)	0.052
Structural urologic conditions‡	2.05 (1.63–2.57)	<0.001		2.15 (1.72–2.68)	<0.001
Neurologic conditions	2.74 (2.13–3.52)	<0.001		2.89 (2.26–3.70)	<0.001
Hospital admissions					
None	Referent	NA		Referent	NA
1–2	1.80 (1.37–2.37)	<0.001		1.74 (1.34–2.25)	<0.001
3–5	3.56 (2.46–5.14)	<0.001		3.50 (2.50–4.88)	<0.001
6–10	4.30 (2.39–7.75)	<0.001		4.34 (2.57–7.66)	<0.001
>10	5.99 (2.08–17.2)	<0.001		6.81 (2.51–18.5)	<0.001
Rheumatologic disease	0.77 (0.57–0.01)	0.061		0.77 (0.59–1.00)	0.050
Kidney disease	1.15 (0.80–1.66)	0.4		NA	NA
Pulmonary conditions	1.00 (0.75–1.34)	0.98		NA	NA
Gastrointestinal conditions	0.86 (0.63–1.16)	0.33		NA	NA
Diabetes mellitus	0.97 (0.72–1.29)	0.81		NA	NA
Cardiovascular disease	0.84 (0.65–1.08)	0.18		NA	NA
No. prescribed drugs	1.03 (1.00–1.05)	0.019		NA	NA
Corticosteroid therapy	0.72 (0.52–0.99)	0.040		NA	NA

*First model included variables that had univariable associations with aerococcal BSI. Age, sex, and county were included in the models but were hidden because matching on those variables prevented appropriate interpretation of their respective estimates. Likelihood-ratio tests and variance inflation analyses were used to guide progressive refinement of the model. BSI, bloodstream infection; NA, not applicable. †Time in months indicates when treatment for another infection occurred before the aerococcal BSI was detected. ‡Included urological malignancies.

**Figure 3:**
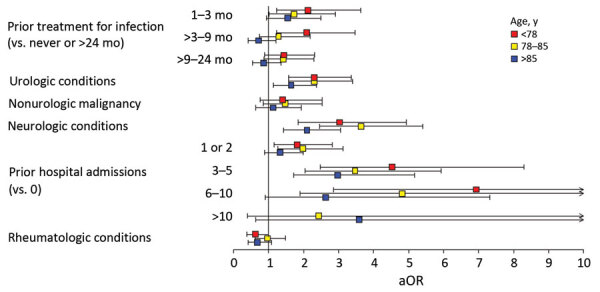
Risk factors stratified by age in nationwide observational case–control study of *Aerococcus* bloodstream infections, Sweden, 2012–2016. Forest plots depicting 3 conditional multivariable logistic regression analyses, 1 analysis each/age tertile. Squares indicate the aOR; error bars indicate 95% CIs. aOR, adjusted odds ratio.

**Figure 4:**
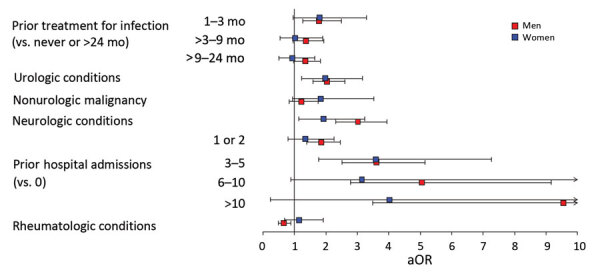
Risk factors stratified by sex in nationwide observational case–control study of *Aerococcus* bloodstream infections, Sweden, 2012–2016. Forest plots depicting 2 conditional multivariable logistic regression analyses, 1 each for male and female patients. Squares indicate the aOR; error bars indicate 95% CIs. aOR, adjusted odds ratio.

In age-stratified models, the associations between aerococcal BSI and hospital admission, previous treatment for infection, malignancy, and neurologic or structural urologic conditions were slightly more pronounced in the lower age tertiles (Figure 3). The sex-stratified models revealed male or female sex did not strongly affect other risk factors for aerococcal BSI (Figure 4).

### Post Hoc Analyses

We performed univariable post hoc analyses according to the primary multivariable modeling. Prescriptions of oral antimicrobial drugs commonly used during 30 days–2 years before aerococcal BSI diagnosis for lower urinary tract infections, such as pivmecillinam, nitrofurantoin, and trimethoprim (odds ratio [OR] 3.02 [95% CI 2.36–3.85]; p<0.001), and upper urinary tract infections, such as ciprofloxacin (OR 3.23 [95% CI 2.55–4.10]; p<0.001) and trimethoprim/sulfamethoxazole (OR 2.76 [95% CI 1.71– 4.40]; p<0.001), were associated with aerococcal BSI (Table 4). Penicillins other than pivmecillinam had only a modest association (OR 1.37 [95% CI 1.11–1.68]; p = 0.003). Clindamycin, macrolides, and tetracyclins were not associated with aerococcal BSI (Table 4).

**Table 4:** Post hoc analyses of risk factors for *Aerococcus* BSIs in nationwide observational case–control study, Sweden, 2012–2016*

Risk factor	Controls, no. (%)	Aerococcal BSI, no. (%)	Odds ratio (95% CI)	p value	Codes†
Prescribed antimicrobial drugs					
Phenoxymethylpenicillin, amoxicillin, or flucloxacillin	512 (22.2)	162 (28.1)	1.37 (1.11–1.68)	0.003	ATC: J01CE02, J01CF05, J01CA04
Macrolides or clindamycin	90 (3.9)	24 (4.2)	1.07 (0.66–1.67)	0.8	ATC: J01F
Tetracyclins	171 (7.4)	49 (8.5)	1.16 (0.82–1.60)	0.4	ATC: J01A
Pivmecillinam, nitrofurantoin, or trimethoprim	201 (8.7)	129 (22.4)	3.02 (2.36–3.85)	<0.001	ATC: J01XE01, J01CA08, J01EA01
Ciprofloxacin	208 (9.0)	140 (24.3)	3.23 (2.55–4.10)	<0.001	ATC: J01MA02
Trimethoprim or sulfamethoxazole	45 (1.9)	30 (5.2)	2.76 (1.71–4.40)	<0.001	ATC: J01EE01
Urologic conditions, including malignancy	753 (31.8)	331 (57.4)	2.88 (2.39–3.47)	<0.001	See Table 1
Renal or ureteral conditions	31 (1.3)	43 (7.5)	5.91 (3.71–9.55)	<0.001	C64–66, D300–302, D410–412, N13, N20, Q60–63, S370–371
Vesical or urethral conditions	40 (1.7)	25 (4.3)	2.57 (1.53–4.24)	<0.001	C67–68, D303–304, D090, D413–414, N21, Q64, S372–373
Prostate and other male reproductive organs	241 (10.4)	123 (27.2)	2.43 (1.89–3.11)	<0.001	C60–63, D074–76, D29, D40, N40–42
Urologic malignancy	152 (6.6)	70 (12.2)	1.96 (1.45–2.63)	<0.001	C51–53, C60–68
Urologic malignancy except prostate	36 (1.6)	15 (2.6)	1.68 (0.89–3.04)	0.094	C51–53, C60, C62–68
Prostate cancer, men	121 (5.2)	58 (10.1)	2.02 (1.45–2.79)	<0.001	C61
Gynecologic cancer, women	5 (1.0)	4 (3.3)	3.27 (0.80–12.6)	0.081	C51–59
Prescription of drugs for prostatic hyperplasia, men	367 (20.2)	130 (28.6)	1.54 (1.23–1.92)	<0.001	ATC: G04C
Urinary tract stones	25 (1.1)	25 (4.5)	4.31 (2.46–7.55)	<0.001	N20–21
Hydronephrosis	10 (0.4)	26 (4.5)	10.8 (5.36–23.7)	<0.001	N13
Malignant disease, nonurologic	182 (7.9)	75 (13.0)	1.75 (1.30–2.31)	<0.001	See Table 1
Lung cancer	2 (0.1)	7 (1.2)	14.2 (3.41–95.2)	<0.001	C34
Skin cancer	112 (4.9)	29 (5.0)	1.04 (0.67–1.56)	0.9	C43–44
Colon cancer	11 (0.5)	5 (0.9)	1.83 (0.57–5.04)	0.3	C18–20
Hematologic malignancy	23 (1.0)	14 (2.4)	2.47 (1.23–4.78)	0.008	C8–9
Neurologic conditions					
Cerebrovascular insult	124 (5.4)	88 (15.3)	3.17 (2.37–4.23)	<0.001	I61–63, I69
Paraparesis or tetraparesis	23 (1.0)	25 (4.3)	4.50 (2.53–8.03)	<0.001	G8
Dementia	122 (5.3)	105 (18.2)	3.99 (3.01–5.27)	<0.001	G30–31, F00–03, F051; ATC: N06D
Parkinson’s disease	58 (2.5)	43 (7.5)	3.12 (2.07–4.67)	<0.001	G20–22; ATC: N04
Multiple sclerosis	4 (0.2)	11 (1.9)	11.2 (3.81–40.5)	<0.001	G35
Estrogen prescription, women	111 (22.5)	23 (18.7)	0.80 (0.48–1.30)	0.4	G03C

*Descriptive analysis of subdivisions of risk factor categories identified in multivariable analyses. ATC, Anatomic Therapeutic Chemical; BSI, bloodstream infection. †Diagnostic codes were derived from the International Classification of Diseases, 10th Revision; drug prescription codes were derived from the ATC classification system (as indicated).

Among urologic conditions, benign prostatic hyperplasia was a common condition, having a modest association with aerococcal BSI (OR 1.54 [95% CI 1.23–1.92]; p<0.001). Prostate cancer (which constituted most urologic malignancies in this group) also correlated with aerococcal BSI (OR 2.02 [95% CI 1.45–2.79]; p<0.001). Diagnoses related to urinary tract stones, especially hydronephrosis (OR 10.8 [95% CI 5.36–23.7]; p<0.001), were rare but strongly associated with aerococcal BSI. Conditions involving kidneys and ureters generally had more robust associations with aerococcal BSI than those involving the bladder and urethra. In women, gynecologic cancers were rare and were not significantly associated with aerococcal BSI (OR 3.27 [95% CI 0.80–12.6]; p = 0.081).

Malignant tumors of the colon and skin did not correlate with aerococcal BSI, whereas lung cancer (OR 14.2 [95% CI 3.41–95.2]; p<0.001) and hematologic malignancies (OR 2.47 [95% CI 1.23–4.78]; p = 0.008) were rare risk factors for this infection. Among neurologic conditions, dementia (OR 3.99 [95% CI 3.01–5.27]; p<0.001), cerebrovascular insult (OR 3.17 [95% CI 2.37–4.23]; p<0.001), Parkinson’s disease (OR 3.12 [95% CI 2.07–4.67]; p<0.001), paraparesis or tetraparesis (OR 4.50 [95% CI 2.53–8.03]; p<0.001), and multiple sclerosis (OR 11.2 [95% CI 3.81–40.5]; p<0.001) were strongly associated with aerococcal BSI.

## Discussion

In this nationwide population-based study in Sweden, we identified associations between aerococcal BSI and urogenital diagnoses in general and urologic malignancy in particular, consistent with previous observations. We also identified associations with neurologic conditions, such as dementia, which has been infrequently reported. Our findings support a considerable predilection for elderly men to acquire aerococcal BSIs and also support the hypothesis that aerococci enter the bloodstream through the urogenital tract and renal mucosa (*24*). That hypothesis is strengthened by the observation that previous prescriptions of antimicrobial drug classes commonly administered for urinary tract infections were positively associated with aerococcal BSI. 

We noted a higher incidence of aerococcal BSI in this study than in previous reports on *A. urinae* and *A. sanguinicola*, although the number of studies estimating rates has been low (*5*,*6*,*12*,*13*,*29*). As in previous studies, we observed sex differences in the tendency toward developing aerococcal BSIs; men had a greater incidence (*5*–*7*,*12*–*14*,*18*,*29*), but the mechanisms of this differential pattern of infection remain unknown. We found male urogenital tract disorders were more common in patients with aerococcal BSI than in controls. We speculate that the disproportionate male prevalence of aerococcal BSI can be explained in part by the preponderance of prostate disorders in elderly men.

The mechanisms of susceptibility to aerococcal BSI in male and female patients warrant further study. Because aerococcal BSIs are more frequent in elderly men, *Aerococcus* spp. have been proposed to enter the bloodstream through the prostate (*3*,*5*–*7*,*12*–*14*,*18*,*29*,*30*). Our findings indicate prostate cancer increases the risk for aerococcal BSI, whereas the association between infection and benign prostate hyperplasia was modest compared with conditions in other structures of the urogenital system. Our results indicate that clinical conditions in the kidneys and ureters were also associated with a higher risk for aerococcal BSI than those in the bladder, prostate, or urethra. A link between neurologic conditions in general, particularly dementia and paresis, and aerococcal BSI has been suggested in a small case series from Denmark, although only for *A. sanguinicola* (*31*). It is possible that a connection between neurologic diseases and aerococcal BSI is mediated by immobility issues in some patients (e.g., cerebrovascular insults leading to immobility) and, in the case of paresis, through catheterization of the urinary tract. Rheumatologic disease and aerococcal BSI had a negative association in multivariable analysis but had a positive crude association; however, the association was relatively modest and borderline significant and might have been a spurious effect of multiple comparisons.

In women, the levels of *A. urinae* and *A. sanguinicola* in vaginal microbiota increase during hormone replacement therapy (*32*). We did not, however, observe an increased risk for aerococcal BSI in women who had been prescribed estrogen medication, although the validity of that finding is limited by the low number of female study participants. One possible explanation is that urogenital carriage of aerococci is less likely to lead to bacteremia in women. That interpretation is consistent with smaller studies in Scotland and southern Sweden in which a larger proportion of men compared with women had *Aerococcus* spp. in blood but not urinary cultures (*11*,*12*,*33*,*34*).

The main strengths of this study are that it is population-based, examined the incidence of and risk factors for aerococcal BSI, and had a follow-up of ≈40 million person-years and a matched control group from the source population, enabling high-precision estimates and robust control for confounding. Access to healthcare in Sweden is not restricted by having private insurance, which might have otherwise introduced bias and possibly caused underestimation of aerococcal BSI incidence and limited the generalizability of our results to persons in lower socioeconomic strata or vulnerable subgroups (*25*). Second, in contrast to previous studies in which species were identified by using varied methods (often involving hospital-based or local inclusion of patients), the participating laboratories used MALDI-TOF mass spectrometry throughout the study period, and we included persons from multiple regions in a population-based manner (*12*–*14*). MALDI-TOF mass spectrometry has been shown to reliably identify *Aerococcus* spp. and has rectified the misclassification of *A. sanguinicola* as *A. viridans* and of aerococci in general as streptococci (*35*). Third, using population-based selection of matched controls enabled relevant comparisons with aerococcal BSIs among persons of the same age, sex distribution, and county of residence. That setup promoted a more efficient analysis of risk factors other than age and sex and reduced potential biases from variabilities in the structure or care registration between counties in Sweden. Moreover, considering the low incidence of aerococcal BSI, we consider it highly unlikely that a substantial proportion of the control population would have had an aerococcal BSI outside of the study uptake area or before the study period. Finally, the use of structured backward selection, according to variance likelihood ratio and variance inflation, enabled additional control for confounding and multicollinearity.

The first limitation of our study is that, despite our approach to control for confounding, residual confounding from factors that were not included in the registers cannot be ruled out. Despite efforts to limit potential sources of bias, claims on causality cannot be made by using a register-based study design. We also acknowledge that our findings might not be generalizable to children and young adults because of the limited number of those participants in this study. Second, information on over-the-counter drugs and drugs given during inpatient care was unavailable; however, except for topical estrogen, no drug reported in this study was available over the counter. Third, iatrogenic immune suppression caused by drugs (other than corticosteroids), especially chemotherapeutics, was not possible to define operationally by using the available registers because those drugs are often administered directly at the clinic and not filled by prescription in a pharmacy outside of the hospital. Thus, the relative effects of treatments for malignancy versus treatments for aerococcal BSI were not possible to evaluate in this study. Fourth, information on intermittent catheterization and the use of medical devices, including permanent urinary catheters, was lacking. Therefore, it was not possible to study the extent to which urinary catheterization might explain the link between neurologic conditions and aerococcal BSI. Finally, *A. urinae* has recently been proposed to comprise a cluster of related species (*36*). Because our study data were collected before that publication appeared, potential clustering was not possible to include in our analyses.

Previous validation studies of inpatient registry data in Sweden have shown that 85%–95% of diagnoses are correct, inasmuch as the ICD-10 code corresponds to the diagnosis entered into the medical records (*27*). However, diagnoses made during primary healthcare are unavailable in the patient register. That lack of accessibility could lead to selection bias, wherein each predisposing condition would only be represented by the stratum of disease severity that justified specialized care, potentially causing low power and inflated OR estimates because milder disease would be undetected. To minimize that risk, we combined registered ICD-10 codes with drug prescription data to capture the conditions of interest more robustly, which is especially critical for conditions such as diabetes mellitus, benign prostatic hyperplasia, and less severe infectious diseases that are generally managed during primary care in Sweden.

We defined aerococcal BSI according to the detection of aerococci in a blood culture. In ≈33% of cases, >1 bacteria species grew in the blood culture. We do not believe that polymicrobial growth reflects contamination of blood cultures by aerococci to any appreciable extent, because they are not known to routinely colonize the skin. It was not possible to determine the relative significance of aerococci when identified with other bacteria; however, we consider the presence of *Aerococcus* spp. in a patient’s blood to represent true bacteremia. A high degree of co-isolation with other established uropathogens, including *E. coli*, *E. faecalis*, and *A. schaali*, also suggests bacteremia originated from a urinary tract infection. Furthermore, prescriptions of antimicrobial drugs commonly taken for urinary tract infections were strongly associated with the development of aerococcal BSI. Whereas that association might reflect a chronic predilection for urinary tract infections, it is also possible that antimicrobial drug treatment promotes ecologic dysregulation, causing aerococcal overgrowth and invasive disease.

In conclusion, our findings substantially strengthen the proposed link between urologic conditions and aerococcal BSI. Neurologic conditions were also strongly associated with aerococcal BSI, although this association might be mediated by bacterial entry through the urothelium. Further study of the mechanisms that underly the associations between urologic and neurologic conditions and aerococcal bacteremia might be especially valuable in light of our findings. Awareness of *Aerococcus* spp. in patients, especially elderly men, will be needed to manage invasive infections.
